# Thromboangiitis obliterans (Buerger's disease)

**DOI:** 10.1186/1750-1172-1-14

**Published:** 2006-04-27

**Authors:** Perttu ET Arkkila

**Affiliations:** 1 Department of Gastroenterology, Helsinki University, Centrala Hospital, 00029 Hus, Finland

## Abstract

Thromboangiitis obliterans or Buerger's disease is a segmental occlusive inflammatory condition of arteries and veins, characterized by thrombosis and recanalization of the affected vessels. It is a non-atherosclerotic inflammatory disease affecting small and medium sized arteries and veins of upper and lower extremities. The clinical criteria include: age under 45 years; current or recent history of tobacco use; presence of distal-extremity ischemia indicated by claudication, pain at rest, ischemic ulcers or gangrenes and documented by non-invasive vascular testing; exclusion of autoimmune diseases, hypercoagulable states and diabetes mellitus; exclusion of a proximal source of emboli by echocardiography or arteriography; consistent arteriographic findings in the clinically involved and non-involved limbs. The disease is found worldwide, the prevalence among all patients with peripheral arterial disease ranges from values as low as 0.5 to 5.6% in Western Europe to values as high as 45 to 63% in India, 16 to 66% in Korea and Japan, and 80% among Ashkenazi Jews. The etiology of thromboangiitis obliterans is unknown, but use or exposure to tobacco is central to the initiation and progression of the disease. If the patient smokes, stopping completely is an essential first step of treatment. The effectiveness of other treatments including vasodilating or anti-clotting drugs, surgical revascularization or sympathectomy in preventing amputation or treating pain, remains to be determined.

## Disease name and synonyms

Thromboangiitis obliterans

Buerger's disease

## Definition

Thromboangiitis obliterans (TAO) is a segmental occlusive inflammatory condition of arteries and veins, characterized by thrombosis and recanalization of the affected vessels [1,2]. It is a non-atherosclerotic inflammatory disease affecting small and medium sized arteries and veins of the upper and lower extremities [3].

## Epidemiology

The disease is found worldwide, but the highest incidence of thromboangiitis obliterans occurs in the Middle and Far East [4,5]. The prevalence of the disease in the general population in Japan was estimated at 5/100,000 persons in 1985 [6]. The prevalence of the disease among all patients with peripheral arterial disease ranges from values as low as 0.5 to 5.6% in Western Europe to values as high as 45 to 63% in India, 16 to 66% in Korea and Japan, and 80% among Jews of Ashkenazi ancestry living in Israel. Part of this variation in disease prevalence may be due to variability in diagnostic criteria [7,8].

## Clinical description

The onset of Buerger's disease occurs between 40 and 45 years of age, and men are most commonly affected. It begins with ischemia of the distal small vessels of the arms, legs, hands and feet. Involvement of the large arteries is unusual and rarely occurs in the absence of occlusive disease of the small vessels [9]. Patients may present with claudication of the feet, legs, hands and arms. The pain typically begins in the extremities, but may radiate to more central parts of the body. As the disease progresses, typical calf claudication and eventually ischemic pain at rest and ischemic ulcerations on the toes, feet or fingers may develop [10]. Due to the likehood of involvement of more than one limb [11], it is advisable to obtain an arteriogram of both arms or legs, or all four limbs in patients who present with clinical involvement of only one limb. Limbs that are clinically not affected could present arteriographic abnormalities. Other signs and symptoms of the disease may include numbness and/or tingling in the limbs, skin ulcerations and gangrene of the digits. Superficial thrombophlebitis and Raynaud's phenomenon occur in approximately 40% of patients with thromboangiitis obliterans [3].

Although Buerger's disease most commonly affects the small and medium-sized arteries and veins in the arms, hands, legs and feet, it has been reported in many other vascular beds. There are case reports of involvement of the cerebral and coronary arteries, aorta, intestinal vessels, and even multiple-organ involvement [12-15].

When TAO occurs in unusual locations, the diagnosis should be made only when histopathological examination identifies the acute-phase lesions [3].

Gastrointestinal involvement of TAO remains rare, however, intestinal manifestations like stricture or perforation of the colon may become apparent long before symptoms of severe peripheral arterial disease in patients with TAO [14].

## Diagnostic criteria

Since the specificity of Buerger's disease is characterized by peripheral ischemia of inflammatory nature with a self-limiting course, diagnostic criteria should be discussed from clinical point of view.

Several different criteria have been proposed for the diagnosis of thromboangiitis obliterans.

### Diagnostic criteria of Shionoya (1998) [16]

• smoking history;

• onset before the age of 50 years;

• infrapopliteal arterial occlusions;

• either arm involvement or phlebitis migrans;

• absence of atherosclerotic risk factors other than smoking.

### Diagnostic criteria of Olin (2000) [10]

• age under 45 years;

• current or recent history of tobacco use;

• the presence of distal-extremity ischemia indicated by claudication, pain at rest, ischemic ulcers or gangrenes and documented by non-invasive vascular testing;

• exclusion of autoimmune diseases, hypercoagulable states and diabetes mellitus;

• exclusion of a proximal source of emboli by echocardiography or arteriography;

• consistent arteriographic findings in the clinically involved and non-involved limbs.

## Diagnostic methods

No specific laboratory test for diagnosing Buerger's disease is available. Unlike other types of vasculitis, in patients with Buerger's disease the acute-phase reactions (such as the erythrocyte sedimentation rate and C-reactive protein level) are normal [3].

Recommended tests to rule out other causes of vasculitis include a complete blood cell count; liver function tests; determination of serum creatinine concentrations, fasting blood sugar levels and sedimentation rate; tests for antinuclear antibody, rheumatoid factor, serologic markers for CREST (calcinosis cutis, Raynaud phenomenon, sclerodactyly and telangiectasia) syndrome and scleroderma, and screening for hypercoagulability. Screening for hypercoagulopathy, including antiphosolipid antibodies and homocystein in patients with Buerger's disease, is recommended.

If a proximal source of embolization is suspected, transthoracic or transesophageal echocardiography and arteriography should be performed. Angiographic findings include severe distal segmental occlusive lesions. The more proximal arteries are normal. The role of modern imaging methods, such as computerised tomography (CT) and magnetic resonance imaging (MRI) in diagnosis and differential diagnosis of Buerger's disease still remains unsettled. In patients with leg ulceration suspected of having TAO, the Allen test should be performed to assess the circulation in the hands and fingers [17].

## Differential diagnosis

The distal nature of TAO and the involvement of the legs and arms help to differentiate this disease from atherosclerosis. In TAO, the internal elastic lamina and the media are preserved in contrast to systemic vasculitis, in which disruption of these lamina is usually striking [18].

An abnormal Allen test [7,19] in a young smoker presenting with leg ulcerations is highly suggestive of TAO. This test demonstrates small vessel involvement in both the arms and the legs. However, an abnormal result can also be present in other types of small vessel occlusive diseases of the hand such as scleroderma, CREST syndrome, repetitive trauma, emboli, hypercoagulable states and vasculitis.

## Etiology

Although the cause of Buerger's disease remains unknown, a strong association with tobacco use has been established [3]. Use or exposure to tobacco plays central role in the initiation and progression of the disease. By using an antigen-sensitive thymidine-incorporation assay, Adar *et al*. [20] showed that patients with TAO have an increased cellular sensitivity to type I and III collagen, compared to that in patients with arteriosclerosis obliterans or healthy males. De Moerloose *et al*. [21] found a marked decrease in frequency of the HLA-B12 antigen in patients with Buerger's disease (2.2% *vs*. 28% in controls). Similarly to other autoimmune diseases, TAO may have a genetic predisposition without a direct "causative" gene mutation. Most investigators feel that Buerger's disease is an immune-mediated endarteritis. Recent immunocytochemical studies have demonstrated a linear deposition of immunoglobulins and complement factors along the elastic lamina [22]. The inciting antigen has not been discovered. The role of hyperhomocysteinemia in the pathogenesis of Buerger's disease is controversial [23]. An association between thrombophilic conditions such as antiphospholipid syndrome and Buerger's disease has also been suggested [24].

Peripheral endothelium-dependent vasodilation is impaired in patients with Buerger's disease, while non-endothelial mechanisms of vasodilation seem to be intact [25].

## Histopathology

While the clinical criteria of TAO are relatively well defined, there is no consensus on the histopathological findings [26]. It is particularly difficult to distinguish morphologically TAO from ateriosclerosis obliterans (ASO). Histopatological findings are also known to vary according to the duration of the disease [3]. The findings are most likely to be diagnostic in the acute phase of the disease, most commonly at biopsy of a segment of a vessel with superficial thrombophlebitis [10]. Other histopathological phases, such as intermediate (subacute) and end-state (chronic) phases, have been described.

The **acute-phase lesions **include an occlusive, highly cellular, inflammatory thrombus with less inflammation in the walls of the blood vessels. Polymorphonuclear leukocytes, microabscesses and multinucleated giant cells may exist. When TAO occurs in unusual locations, the diagnosis should be made only when histopathological examination identifies the acute-phase lesion.

In the **intermediate phase **of disease there is progressive organization of the thrombus in the arteries and veins.

When only organized thrombus and fibrosis are found in the blood vessels, the phase is considered to be **end-stage **[27-29].

## Management including treatment

The most effective treatment for Buerger's disease is smoking cessation. It is therefore essential that patients diagnosed with Buerger's disease stop smoking immediately and completely in order to prevent progression of the disease and avoid amputation [3,30]. Early treatment is also important, because Buerger's disease may provoke social problems that influence quality of life [31]. Even smoking one or two cigarettes per day or using smokeless tobacco (chewing tobacco or using nicotine-containing patches) may keep the disease active [32,33]. If there is no gangrene when the patient discontinues smoking, amputation is avoided. Patients who continue smoking are at risk of amputation of fingers and toes. Physicians must educate and counsel their patients repeatedly about the importance of discontinuing the use of all tobacco products.

Despite the very strong correlation between smoking cessation and the decline of clinical manifestations of TAO, patients may continue to have claudication or Raynaud's phenomenon after complete cessation of tobacco usage [3].

Supportive care should be directed towards maximizing blood supply to the affected limbs. Care should be taken to avoid thermal, chemical or mechanical injury, especially from poorly fitting footwear or minor surgery of digits, as well as fungal infection. Vasoconstriction provoked by cold-exposure or drugs should be avoided.

Despite the clear role of inflammation in the pathogenesis of TAO, anti-inflammatory agents, such as steroids, have not been shown to be of real benefit. The results of intravenous therapy with Iloprost (a prostaglandin analogue) show that this drug is superior to aspirin in providing total pain relief at rest and complete healing of all trophic changes. It diminishes the risk of amputation [34]. Although acetylsalicylic acid (aspirin) is often prescribed to patients with Buerger's disease, the benefit of this or other orally administered anti-clotting agents has not been confirmed by controlled studies. Intra-arterial thrombolytic therapy with streptokinase has been tested in some patients with gangrene or pregangrenous lesions of the toes or feet, with some success in avoiding amputation [35].

For patients with TAO, arterial revascularization is usually not possible due to the diffuse segmental involvement and distal nature of the disease [3]. The benefit of bypass surgery to distal arteries also remains controversial because of the high incidence of graft failure [36]. However, if the patient has severe ischemia and there is a distal target vessel, bypass surgery with the use of an autologous vein should be considered [37-39].

Sympathectomy may be performed to decrease arterial spasm in patients with Buerger's disease. A lapraroscopic method for sympathectomy has also been used [40,41]. Sympathectomy has been shown to provide short-term pain relief and to promote ulcer healing in some patients with Buerger's disease, but no long-term benefit has been confirmed [40]. Spinal cord stimulator and vascular endothelial growth factor gene therapy have been used experimentally in patients with Buerger's disease with promising results [42,43].
